# 100% Classification Accuracy Considered Harmful: The Normalized Information Transfer Factor Explains the Accuracy Paradox

**DOI:** 10.1371/journal.pone.0084217

**Published:** 2014-01-10

**Authors:** Francisco J. Valverde-Albacete, Carmen Peláez-Moreno

**Affiliations:** 1 Departamento de Lenguajes y Sistemas Informáticos, Universidad Nacional de Educación a Distancia, Madrid, Spain; 2 Signal Theory and Communications Department, University Carlos III Madrid, Madrid, Spain; Università degli Studi di Milano (University of Milan), Italy

## Abstract

The most widely spread measure of performance, accuracy, suffers from a paradox: predictive models with a given level of accuracy may have greater predictive power than models with higher accuracy. Despite optimizing classification error rate, high accuracy models may fail to capture crucial information transfer in the classification task. We present evidence of this behavior by means of a combinatorial analysis where every possible contingency matrix of 2, 3 and 4 classes classifiers are depicted on the entropy triangle, a more reliable information-theoretic tool for classification assessment.

Motivated by this, we develop from first principles a measure of classification performance that takes into consideration the information learned by classifiers. We are then able to obtain the entropy-modulated accuracy (EMA), a pessimistic estimate of the expected accuracy with the influence of the input distribution factored out, and the normalized information transfer factor (NIT), a measure of how efficient is the transmission of information from the input to the output set of classes.

The EMA is a more natural measure of classification performance than accuracy when the heuristic to maximize is the transfer of information through the classifier instead of classification error count. The NIT factor measures the effectiveness of the learning process in classifiers and also makes it harder for them to “cheat” using techniques like specialization, while also promoting the interpretability of results. Their use is demonstrated in a mind reading task competition that aims at decoding the identity of a video stimulus based on magnetoencephalography recordings. We show how the EMA and the NIT factor reject rankings based in accuracy, choosing more meaningful and interpretable classifiers.

## Introduction

Classification is an ubiquitous task in Science, Technology and the Humanities [1]. Usage ranges from diagnosing diseases [2] or the status of tumors using gene expression data [3] to the actual classification of tumor classes [4]; from analyzing human performance in perceptual tasks [5] to analyzing that of automated remote sensors [6] or automatic speech recognition machines [7]. If follows that the assessment of the performance of classification processes is of paramount importance for Scientific, Technological and Societal reasons [1], [8]–[10].

To set the theoretical backdrop for our discussion, consider a set of 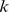 *prior, instance or true classes* 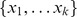 and a discrete random variable 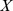 distributed according to a *prior class distribution* 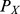. Consider also a set of 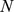 *instances or patterns*, each belonging to only one of those classes, but we do not know precisely which. A *classification* is a process whereby each of those instances is assigned to one among a set of 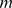 *decision or predicted classes* 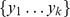 generating a discrete random variable 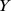 distributed according to a *posterior class distribution,* 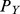, so that the joint events of this classification process consist of “presenting one instance of an input class 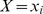 for classification and deciding the output class to be 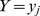
”.

**Figure 1:**
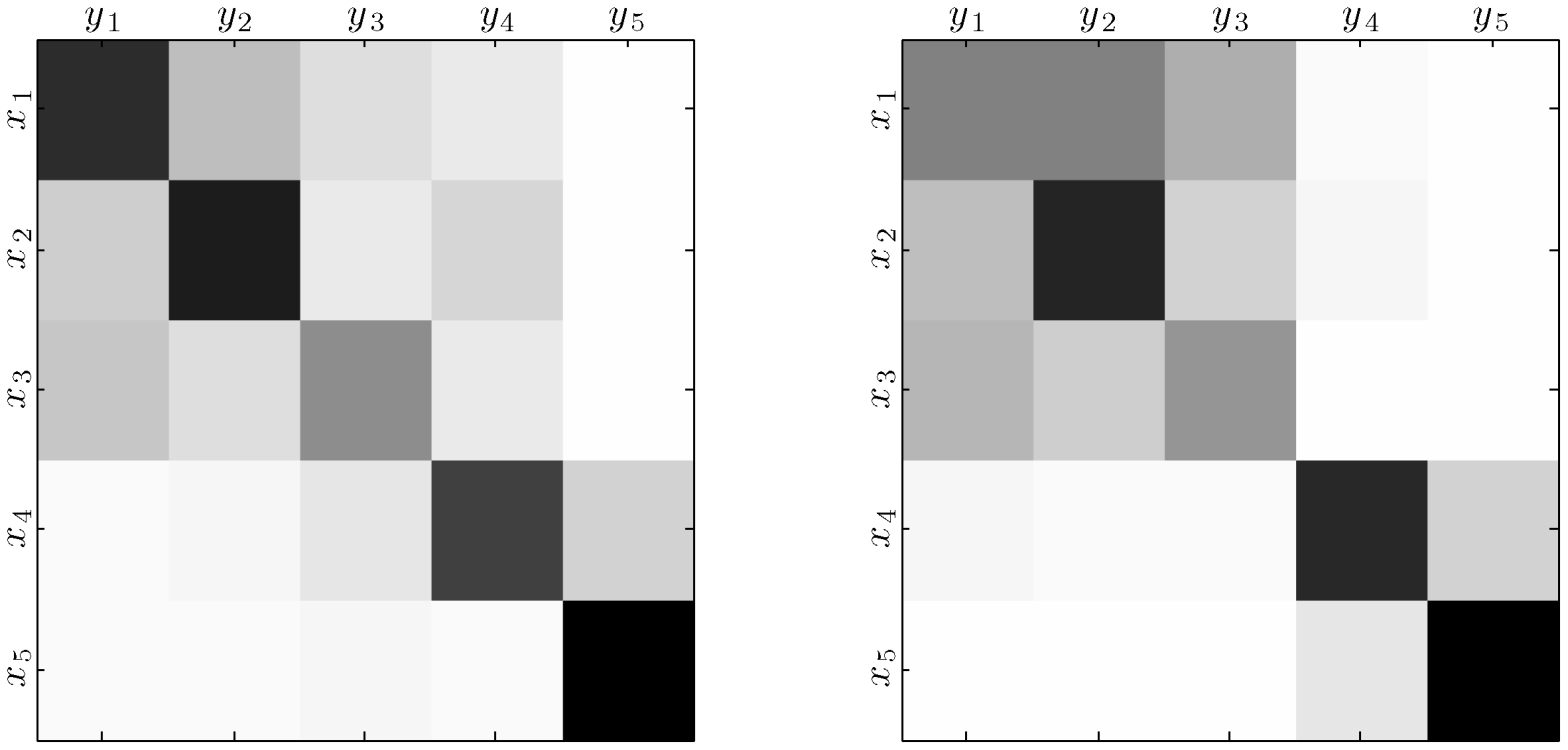
Heatmap of the best classifiers of the MEG mind reading competition [23] according to accuracy (left) and the EMA and the NIT factor (right) criteria. Rows correspond to stimulus 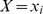 and columns to the decision 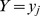 or response. Darker hues correlate with higher joint probability 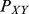. The heat map on the left reveals that the best classifier according to accuracy does not capture the fact that stimuli 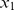, 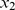 and 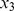 belong to a particular category whilst 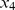 and 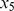 belong to another. A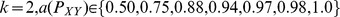 B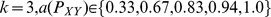 C
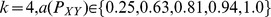

To measure the performance of the classification process we use its *confusion matrix*, a special contingency table 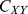 counting the occurrences of the joint events. Usually, the maximum likelihood estimate of the joint probability 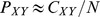 is used as summary data. Figure 1 represents two such contingency matrices for a *brain decoding* or *mind reading* task consisting in automatically identifying the class of video stimulus shown to the subjects based on magnetoencephalography (MEG) data. Five different types of stimuli were presented: the first three ones (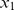, 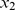 and 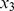) belonging to the category of *short* clips (6–26 s. long) and the last two (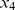 and 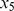) to the category of *long* clips (approximately 10 min. long).

Performance evaluation takes the form of the exploratory analysis of this confusion matrix or joint distribution. For instance, the *de facto* standard for performance *visualization* for binary—that is, two-class—classification is the Receiver-Operating-Characteristic (ROC) [11], but its generalization to higher class numbers is not as effective. We have argued elsewhere that the De Finetti entropy triangle (ET) [12] is a better tool to analyze classifier performance, with a solid information-theoretical basis, and not plagued with the problems of the ROC—see *sec:mms: sec:entropy-triangle*. In any case, neither device provides a *single* figure-of-merit or performance measure to compare systems, a practice cherished by researchers.

As a single figure-of-merit, by far the most widespread performance criterion used is *accuracy*, defined as the fraction of correctly classified instances, 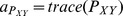. This is probably due to its easy and intuitive nature, despite many reasons *not* to do so [13]. In [14], this and many other performance measures were examined in the context of several machine learning tasks, but inconclusive results as to their fitness of purpose were reached. However, the comparison made evident that accuracy was one of the measures that possessed the least number of invariants with respect to changes in confusion matrix entries, a detrimental quality. An earlier paper [15] had already argued for the factoring *out* of the influence of prior class distributions on similar measures.

It is now acknowledged that *high accuracy is not necessarily an indicator of high classifier performance* and therein lies the *accuracy paradox* [16]–[18]. For instance, in a predictive classification setting, predictive models with a given (lower) level of accuracy may have greater predictive power than models with higher accuracy. This deleterious feature is explained in-depth in Section *sec:crit-accur-using*. In particular, if a single class contains most of the data, a *majority classifier* that assigns all input cases to this majority class (the one concentrating the probability mass of 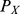) would produce an accurate result. Highly *imbalanced* or *skewed* training data is very commonly encountered in samples taken from natural phenomena. Moreover, the classes' distributions of the samples do not necessarily reflect the distributions in the whole population since most of the times the samples are gathered in very controlled conditions. This skewness in the data hinders the capability of statistical models to predict the behavior of the phenomena being modeled and data balancing strategies are then advisable [19].

In this paper, we claim that performance measures based in the statistical information transfer from 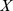 to 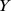 may be better measures for classification if *predictive classification error is not the paramount performance criterion*. This is the case, for example, of classifiers not used to make final decisions but, instead designed to be components of more complex diagnostic systems (as in [19]) or when the conditions in the experimentation stage during which the data is collected do not hold in the deployment stage, as mentioned before. For this purpose, in Section *sec:perpl-its-prop* we establish the basis of our analysis in the propagation of *perplexity*—the effective number of classes a classifier sees—a concept that is directly related to accuracy.

In Section *sec:perf-meas-based* we use the *remaining input perplexity* 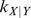 to claim that the *entropy-modulated accuracy (EMA)*, defined in (3), is a better measure of classifier performance than accuracy for several reasons: it is well-grounded in information-theoretical terms, it provides an intuitive interpretation of the statistical learning process as the transfer of the information from the phenomena that are being modeled over a virtual channel, it factors out the influence of the input and output class distributions, it is invariant to permutations in the columns of the confusion matrix enabling the identification of cross-labeling errors common in unsupervised learning methods, and it is a pessimistic estimate of accuracy. For the same reasons, the *normalized information transfer factor ( NIT factor )*, defined as in (5), adds to some of the previous advantages the fact that it is capable of assessing the effectiveness of the learning process in the classifier, it is co-variant with expected mutual information (MI) [20], and contra-variant with the variation of information [21].

In *sec:example-use*, we suggest how to apply these metrics to a classification task, instantiating the process for a mind-reading challenge using multi-classification on magnetoencephalography signals, that shows one clear instance where ranking by EMA and NIT factor provides a more interpretable classifier than accuracy-based ranking. We provide further evidence, examples and a comparison with other metrics in *File S1*. The paper is closed with a *sec:discussion* where we also compare EMA and the NIT factor with two previously proposed measures for classification assessment and show the superiority of our proposal.

## Results

### A critique of accuracy using information-theoretic principles

**Figure 2:**
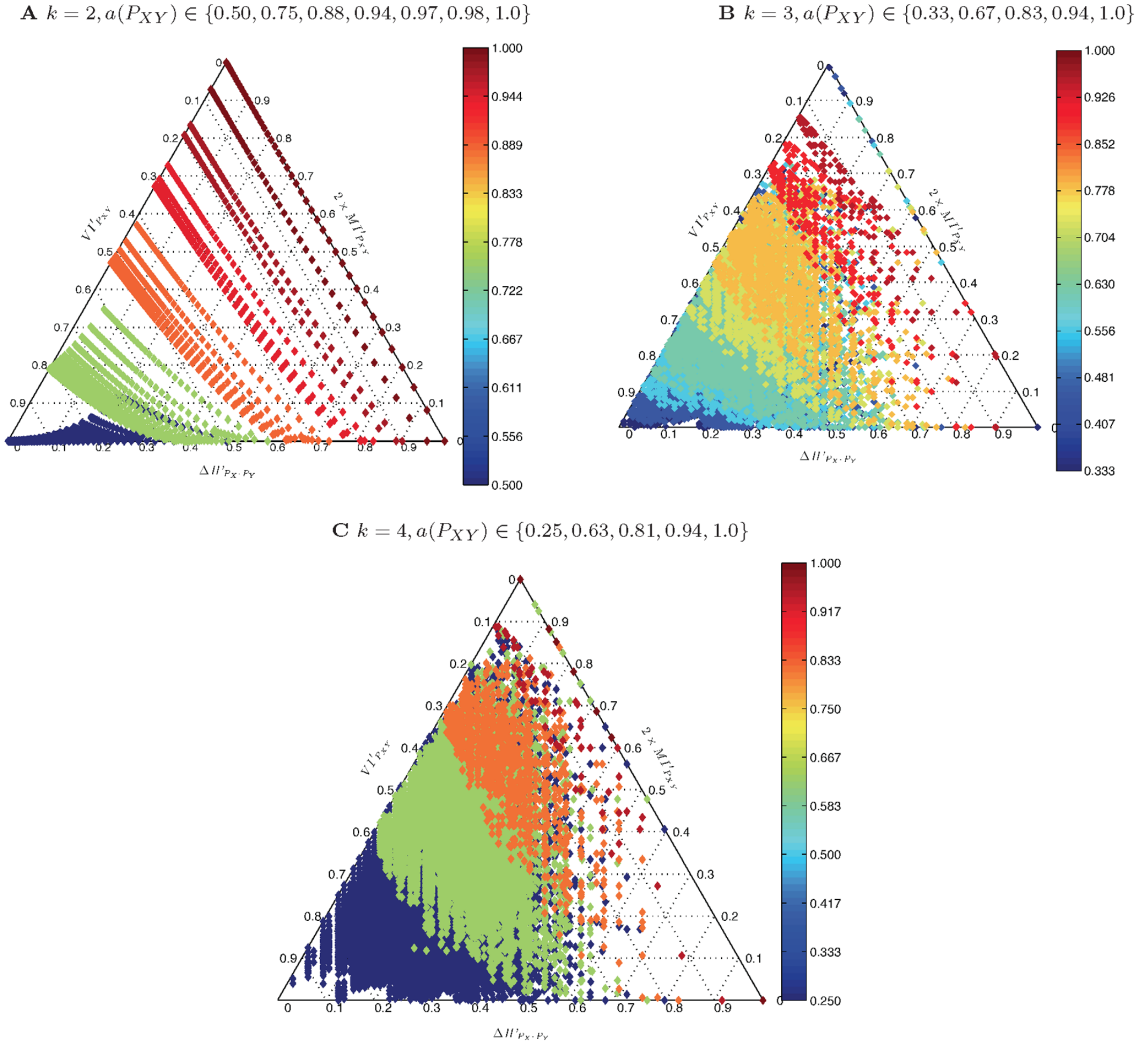
(Color online) Entropy decomposition for square matrices of (A) 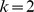, (B) 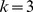, and (C) 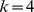 (decimated), representing confusion matrices for a classification task at different accuracy levels as described by the right color bar. The interspersing of the plots representing matrices with different accuracies but similar entropies is evident at all levels for 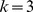 and 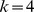 but only for lower levels of accuracy for 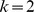. This entails that accuracy is not a good criterion to judge the flow of information from the input labels to the output labels of a classifier (see text).

To assess the theoretical adequacy of accuracy, we generated some samples of the space of joint count distributions for 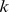 input and output classes and 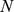 instances of classification with a prescribed accuracy (see Section *sec:datasets* for the details). Then, their entropy decomposition was calculated and plotted in the ET (see Section *sec:mms, sec:entropy-triangle*). Figure 2 presents the cases 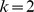 with 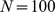, 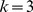 with 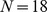 and 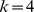 with 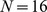.

A number of observations can be gleaned from this figure:

- *Matrices of a particular accuracy level are interspersed with those of many other accuracy levels*. This phenomenon is the more prevalent the lower the accuracy level, although the behavior differs for different 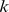. For 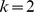 interspersing ends for accuracies over 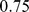 while for 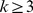 it spreads to the whole range 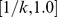.
- *For every prescribed accuracy level, the normalized mutual information ranges in* 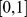, that is, there are matrices with accuracy over 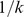 transmitting little or no information. This is the case even for high-accuracy matrices, including those with accuracy 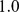.
- Conversely, matrices with different accuracy may exhibit the same normalized mutual information, for instance, check at 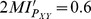.
- There is an accumulation of distributions with high entropy (low 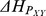 values, left side of ET), as predicted by theory [22].

We are driven to conclude that accuracy is not a trustworthy criterion to judge the degree to which a particular classification process transfers information from the input class distribution to the output decision class distribution.

### Perplexity and its propagation in multiclass classifiers

The question poses itself whether it is possible to conjoin accuracy and mutual information transfer in a single measure. To provide an affirmative answer to this we first state the hypothesis:

#### Hypothesis 1

In the absence of information about the items distributed according to a uniform prior class distribution, a classifier is expected to guess correctly 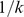 of the times.

We will show that the EMA amounts to a ‘pessimistic’ accuracy estimate according to this hypothesis. For the sake of generality, suppose that the cardinality of the set of atomic events of 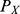 is 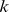 and that of 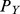 is 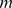. Classification tasks with uniform input class distributions are often called *balanced* or *unskewed*. Let us denote this uniform input distribution as 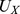 and accordingly, 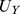 will represent a uniform distribution of the outputs. Now 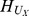 and 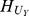 represent the entropies of 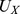 and 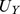 respectively. Then 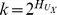 and 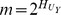, so 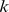 is a measure of the *theoretical perplexity* of a classifier in a balanced task, that is, the number of *possible* events.

By analogy, call 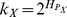 and 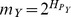 the *perplexities* of variables 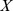 and 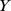 respectively. They are in fact an estimation of the *effective*—as opposed to the *possible*—number of atomic events behind 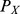 and 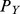. Note that 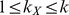 and 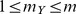 and that 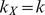 (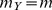) precisely when 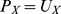 (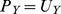). Similarly, 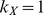 (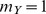) when 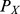 (resp. 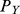) resembles a Kronecker delta function—that is, the input (and output) distribution is utterly skewed towards one class.

If we now define the quotient 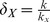 (respectively, 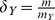) we can see that
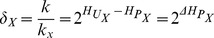


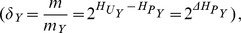
where 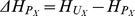 ( 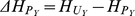.) We interpret this quantity as the decrement (increment) in perplexity due to the choice of input (output) marginals of 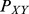.

The most important concept in our discussion is the *information transfer factor* 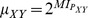: if we introduce two new *remaining perplexities*, 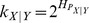 and 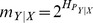, from the well-known formulae 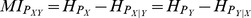 this crucial quantity can be understood as the perplexity variation of 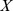 and 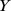 produced by the subtraction/addition of their mutual information,
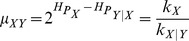


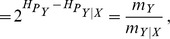
hence the name.

It is easy to see that we have completed two different, sequentially related, decompositions of the perplexity of the variables,
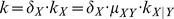
(1)

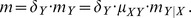



**Figure 3:**
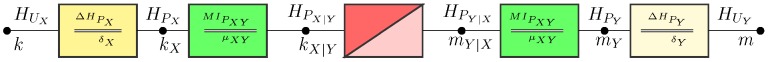
(Color online) Entropy (above) and perplexity (below) decomposition chains for a joint distribution. Left, perplexity reduction in the input (learning) chain; right, perplexity increase in the output chain, related to classifier specialization. The colors refer to those of Fig. 5.(B). The ordering of the boxes is a convention to reveal the prior and posterior natures of the perplexities of class distributions.

This proves that an alternative way of conceptualizing the flow of information from one variable to the other is in terms of increments or decrements of their perplexity instead of the flows of entropies, as depicted in Fig. 3. In fact, the following inequalities can easily be checked,
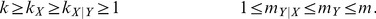
(2)


Note that analogue decompositions for marginal entropies were introduced in [12], and are here collected as *sec:mms: sec:split-entr-triangle*. We will see next how this conceptualization allows us to devise an alternative to accuracy where the decomposition of equation (1) underlines the preeminence of 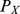 for assessing performance.

### Two performance measures based on perplexity

Consider a confusion matrix for a classifier obtained from 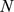 instances of classification pairs. The lowest accuracy is that of a classifier returning a uniform count matrix: the most balanced testing dataset will distribute 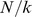 to each class and a clueless classifier will further redistribute these uniformly to each output class as 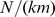 instances. Since the diagonal has 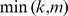 cells, the diagonal sum is
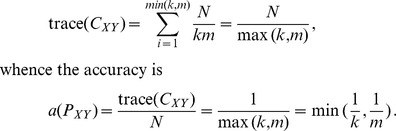



It is bounded by 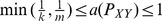 and any value smaller than the lower bound is an sure indication that a permutation of the output tags will ensure higher classification accuracy, that is, a better mapping of input to output *class names*.

Consider the perplexity reduction chain of Fig. 3. To the extent that the number of input classes and their distribution is a given—whereas 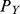 is a construct of the classifier—we want to concentrate on measuring how well the input class distribution was learned by the training process, that is, in the prior class distribution perplexity reduction of equation (1). Regarding the classifier training algorithm, 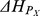 is a given and cannot be modified, whereas 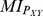 quantifies the amount of *successfully* learned information. More importantly for our purposes, 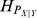 is the amount of information the classifier *failed* to learn. Therefore the EMA appears naturally as a quality measure based in the remaining perplexity of the 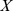 variable
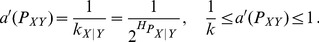
(3)


Since 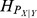 is the entropy of 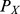 ignored by the classifier, as per our hypothesis and in the absence of any other source of information *this is the expected performance of the classifier with equivalent (possibly fractional), equally likely* 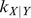 *classes*: the higher this number, the worse the classifier will be.

To illustrate this, notice that when the training process of the classifier has been able to capitalize on all mutual information to leave no remaining perplexity, 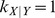, whence 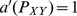. Similarly, 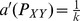, either because the classifier has utterly failed to capture any information between 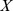 and 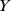, 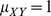, or because the entropy of the data was minimal, 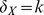.

Notice that when the entropy of 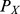 is not maximal 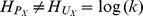 then 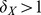 whence 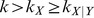 and the EMA detects an *artificial* lower bound for (2), 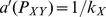. The artifice here is that this increase does not depend on the training of the classifier but on the prior class distribution. This suggests including a correction into equation (3) to account for the deviation from uniformity in the prior class distribution 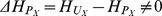 so that
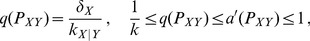
(4)with 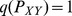 when both 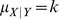 and 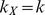, implying that 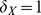 and 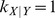. Note that 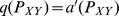 if and only if 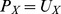. Unlike the case of 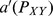, the eventuality that the data are not uniformly distributed is corrected on 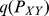, as 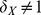 entails 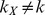. Moreover, the further away from a uniform prior class distribution to the classifier, the worse its upper range bound will be. Eventually, for 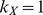
—which implies 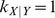 by equation (2) whence 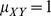
—we have, again, the worst possible value of the measure, 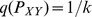. Notice that in this accuracy-optimal case 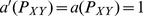, but in an unhelpful way. Essentially, making the input data less random impacts the ability of the classifier to capitalize in mutual information to bind together input and output, and this is registered by the measure. The *normalized information transfer factor* can be rewritten as,



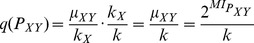
(5)Note also that NIT factor does *not* depend directly on the input or output distribution. Conveniently, since the normalized information transfer factor is a monotonic function of normalized mutual information the relative height in the ET offers a visual tool to quickly inspect such effectiveness. Finally, when evaluating a set of systems in the same task, 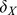 is constant throughout the evaluation, so 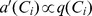, and they offer the same ranking results, easily visualized in the ET.

For the reasons above, we posit the EMA in (3) to measure the performance of classification tasks, and the NIT factor in (4) or (5) to measure the effectiveness of the classifier learning process.

### Assessing classifiers with EMA and the NIT factor

**Figure 4:**
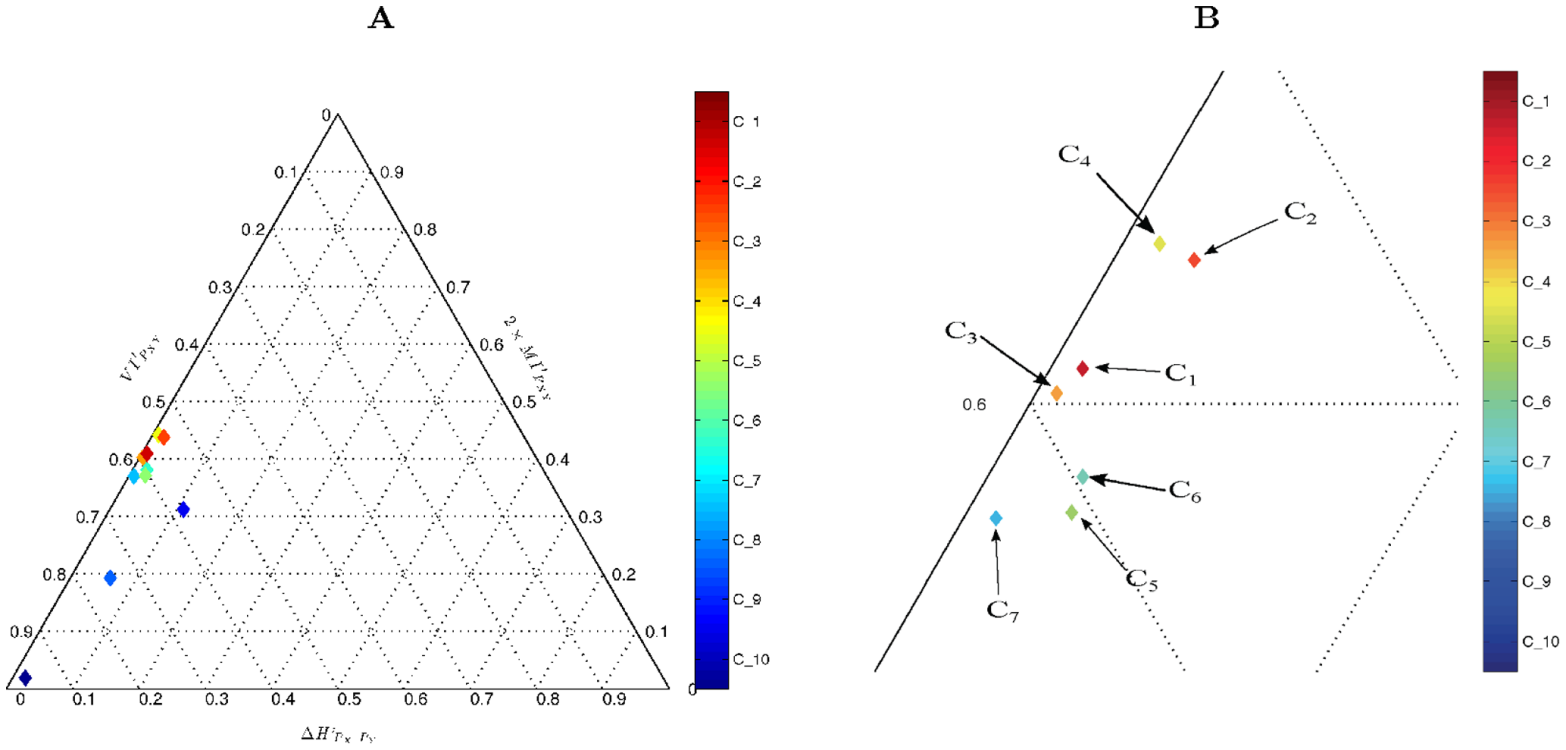
(Color online) Entropy triangle for the MEG mind Reading data ordered after accuracy (A) and a detail of the participants of higher accuracy (B). The ranking following accuracy is at odds with the EMA and the NIT factor ranking based in mutual information (height, right scale of triangle). The detail in (B) shows that participant 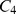, closely followed by 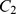 should have been ranked first after this criterion.

In this Section we present an example of how to use the EMA and the NIT factor in automatic classifier evaluation tasks. We consider the case of the MEG mind reading challenge organized by the PASCAL (Pattern Analysis, Statistical modeling and ComputAtional Learning) network [23]. Since accuracy was the “official” evaluation criterion, for comparison purposes Fig. 4.fig: (A) presents the results in the entropy triangle ordered by accuracy as reflected in the coloring of the points. System 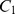 at 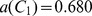 was deemed the winner with 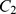 close behind at 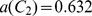. In a detail of the dense region of harder competition in Fig. 4.(B) clusters 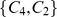, 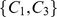 and 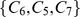 are evident. We next suggest a procedure to analyze the classification performance of a population of classifiers:

1. **Use** 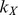 **to assess the effective number of classes of the data.** At 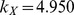 down from 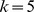, the task is quite balanced, guaranteeing that systems will find it harder to specialize as majority classifiers.
2. **Use EMA to rank classifiers.** Table 1 presents the perplexities, accuracies, the EMA and the NIT factor for the confusion matrices of the classifiers that took part in the task. Ranking 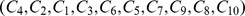 suggests itself, aligned with increasing mutual information (right axis). Indeed, after EMA, 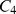 should have been the winner of the competition, followed closely by 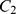.
3. **Use the ET to individually assess each classifier.** From the ET diagram it is evident that those classifiers with highest mutual information and accuracy—the first seven classifiers—are not specialized while classifier 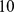, and, to a lesser extent, 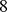 and 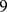 are. The worst classifier is barely above random at 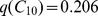.
4. **Use the NIT factor to assess whether the population of classifiers has solved the task.** Overall, for the top ranked classifier we have 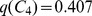, showing that the task has indeed not been effectively solved by the participants, either individually or collectively.

**Table 1:** Perplexities, accuracy (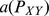), *EMA* (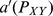) and *NIT factor* (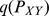) for MEG Mind Reading confusion matrices ranked by accuracy.

Exp.	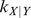	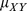	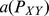	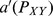	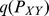
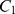	2.562	1.932	0.680	0.390	0.386
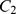	2.447	2.023	0.632	0.409	0.405
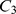	2.589	1.912	0.628	0.386	0.382
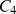	2.430	2.037	0.622	0.412	0.407
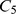	2.723	1.818	0.565	0.367	0.364
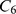	2.682	1.846	0.542	0.373	0.369
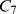	2.730	1.813	0.539	0.366	0.363
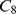	3.629	1.364	0.472	0.276	0.273
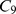	2.995	1.653	0.443	0.334	0.331
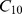	4.801	1.031	0.242	0.208	0.206

Class 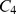 should have been ranked above the rest by EMA or NIT factor (in all cases 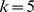 and 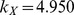).

The result of this process is an assessment of a (population of) classifiers, whereby one may discuss the advantages of EMA and NIT factor vis–vis other performance measures, for instance, accuracy. Further examples of using this procedure to evaluate classification tasks can be found in the *File S1*.

#### EMA and NIT factor vs. Accuracy

The authors of the report on the MEG Mind Reading challenge attempted an analysis of the ranking results and specifically compare classifier 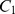 to 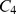 since the heat map of the latter seems to be “cleaner” [23] (see Fig. 4 with the heat map of 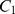 (left) to 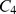 (right)). For them, classifier 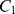 essentially came out first because it used the “learning capacity” of its technique to improve classification error while 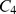 used the capacity to better distinguish the two categories of classes present in the task (with stimuli 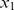 to 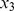 belonging to a first category whilst 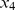 and 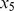, to another) but was worse at capturing the distinctions among the classes of the first category.

Our rejection of this judgement comes from believing that the goal of recovering class structure is as worthy as minimizing classification errors. The interpretability of the results of 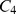 is superior to those of 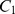 since it has better captured the nature of the underlying phenomenon. This means that the errors committed by 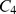 are likely to be inside the same category of the correct response (given the nearly block diagonal structure of its heat map) while in the case of 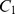, for example, the probability of having stimuli of the first category erroneously predicted as 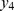 is very high.

The EMA and the NIT factor prove apt at considering the value of representing the underlying structure with their tight relation to perplexity. In fact, according to [23], while 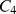, 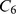 and 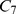 focused on solving the so-called *domain adaptation problem*—the mismatch in training and testing conditions—with advanced machine learning techniques, many of the other teams, including 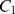, addressed it by placing more weight on the labeled *test* samples provided along with the *train* samples, when validating the learned classifier, thus *explicitly* boosting test set accuracy.

## Discussion

### Measure definition

Perplexity has already been used as a performance measurement for language modeling where it refers to the expected average of alternatives a model has at every word history [24]. It is also often used as an off-line method for speech recognition task evaluation following the intuition that a classifier using a lower-perplexity model will outperform a higher-perplexity one, all other things equal.

It cannot be stressed enough that since the EMA and the NIT factor concentrate in the prior class distribution and mutual information, it is harder for classifiers to boost their performance by manipulating the posterior class distribution through specialization: only the increase in information transfer through 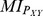 will improve the evaluation figure.

Considering robustness, the EMA, being a harsher, worst-case criterion, might be more deserving of trust than easygoing and unreliable accuracy to, for instance, guide decision making. It certainly has a more interpretable and less easily bendable criterion—specially if *reporting* the classification error is not the ultimate goal. Furthermore, in cases where 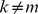
—for instance, when using a “reject” class—the EMA and the NIT factor are still defined, whereas accuracy is problematic, and not very much used.

### Classification task assessment

As seen in the MEG mind reading example, the EMA and the NIT factor are capable of determining whether a task has been effectively solved or not. But it cannot distinguish whether this is caused by technical limitations in the classifier selection process or because the task is inherently “hard”. Only the kind of iterated classification effort of community research that attempts many different classifier-building techniques on the same task can be effective for this purpose.

Nevertheless, the effective input perplexity 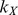 can ensure that, methodologically at least, the task is “as hard as it should be” at 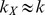. Furthermore, our developments show clearly that a failure to maintain prior class distribution uniformity in the design or capture of the task data entails that the expected mutual information—therefore the NIT factor —captured by any possible classifier that solves the task can never reach maximal levels. This is a strong guideline for prospective collectors of datasets, although data balancing strategies after data collection can also be used to achieve this goal [19].

### Measure comparison

Several other measures have sprouted to deal with the inadequacies of accuracy such as the Area-Under-the-(ROC)-Curve [8], [25], the Variation of Information [21], the Relative Classifier Information [26], the Confusion Entropy [10], [27] or Cohen's Kappa [13], but their use is not widespread, specially for the non-binary case, due to complexity of calculation, disparate purposes or each measures' own shortcomings. For instance, the AUC first needs to find a (multiclass) ROC representation of the task by obtaining multiple classifiers, possibly with the help of a parameter in the classifier learning process. The trading for good-vs-wrong decisions in terms of the parameter can then be judged from the Area-Under-the-ROC curve, which is then a measure *on the learning method or model*. In contrast, EMA would provide a different point in the ET for each classifier whence the best of these classifiers could be chosen. Complementarily, on the *population of classifiers*, a statistical description of the NIT factor could be used to assess the learning capabilities of the method.

In classification proper, to illustrate the disparity of the conclusions that can be reached with alternative performance measures, we have included in *File S1* a comparison of the classical Matthew Correlation Coefficient (MCC) [28] and the Confusion Entropy (CEN) [27]—whose similarities are also explored in [10]–on three different classifications tasks: the MEG Mind Reading task already explored, the TASS sentiment analysis task [29]–both machine learning tasks—and the well-known Miller & Nicely human perceptual capability exploration task [5].

For each task we provide the heat maps of the confusion matrices (Figs. S1, S2 and S4 in *File S1*) as customary. We also provide the tables detailing perplexities, EMA, NIT factor, 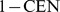 and MCC' related values (Tables S1, S2 and S3 in *File S1* ). The entries in the tables are ordered by accuracy. For the TASS and M&N data we also supply the ET's with the color bar according to EMA, 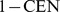 and MCC' (Figs. S3 and S5). Their comparison, detailed in the *File S1* Section, reveals that MCC' is highly correlated with accuracy in ranking results and shows similar shortcomings. Even though CEN performs a little better, it is highly biased towards majority classifiers providing over optimistic assessment for them. Notably, once the ET, EMA and the NIT factor have shed light on the problem, reassessment of prior evidences for either CEN or MCC prove them not to be so advantageous in evaluating classifiers.

## Materials and Methods

### The entropy triangle

**Figure 5:**
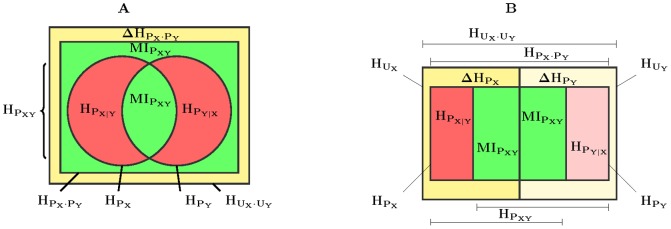
(Color online) Extended information diagrams of entropies related to a bivariate distribution: (A) conventional diagram, and (B) split diagram. The bounding rectangle is the joint entropy of two uniform (thence independent) distributions 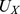 and 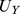 of the same cardinality as 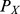 and 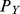. The expected mutual information 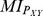 appears *twice* in (A) and this makes the diagram split for each variable symmetrically in (B).

Consider two discrete random variables 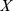 and 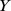 and their joint probability distribution 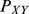. An entropy diagram somewhat more complete than what is normally used for the relations between their entropies was presented in [12] and is here depicted in Fig. 5(A). We distinguish in it the familiar decomposition of the joint entropy 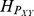 as the two entropies 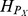 and 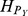 whose intersection is 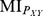. But notice that the increment between 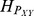 and 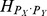 is yet again 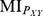, hence the expected mutual information appears *twice* in the diagram. Further, the interior of the outer rectangle represents 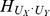
—with 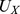 and 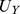 the uniform distribution on inputs and outputs—,the interior of the inner rectangle 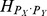, and 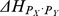 is their difference. Finally, the *variation of information* 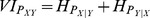 was found to be an important quantity in [21]. Putting together this information results in the *balance equation for information related to a joint distribution*,
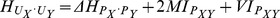
which can be further normalized in 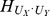,
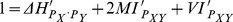
(6)and represented in a De Finetti or ternary diagram as the equation of the 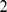-simplex in normalized 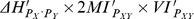 space, hence the name entropy triangle, *ET*.

**Figure 6:**
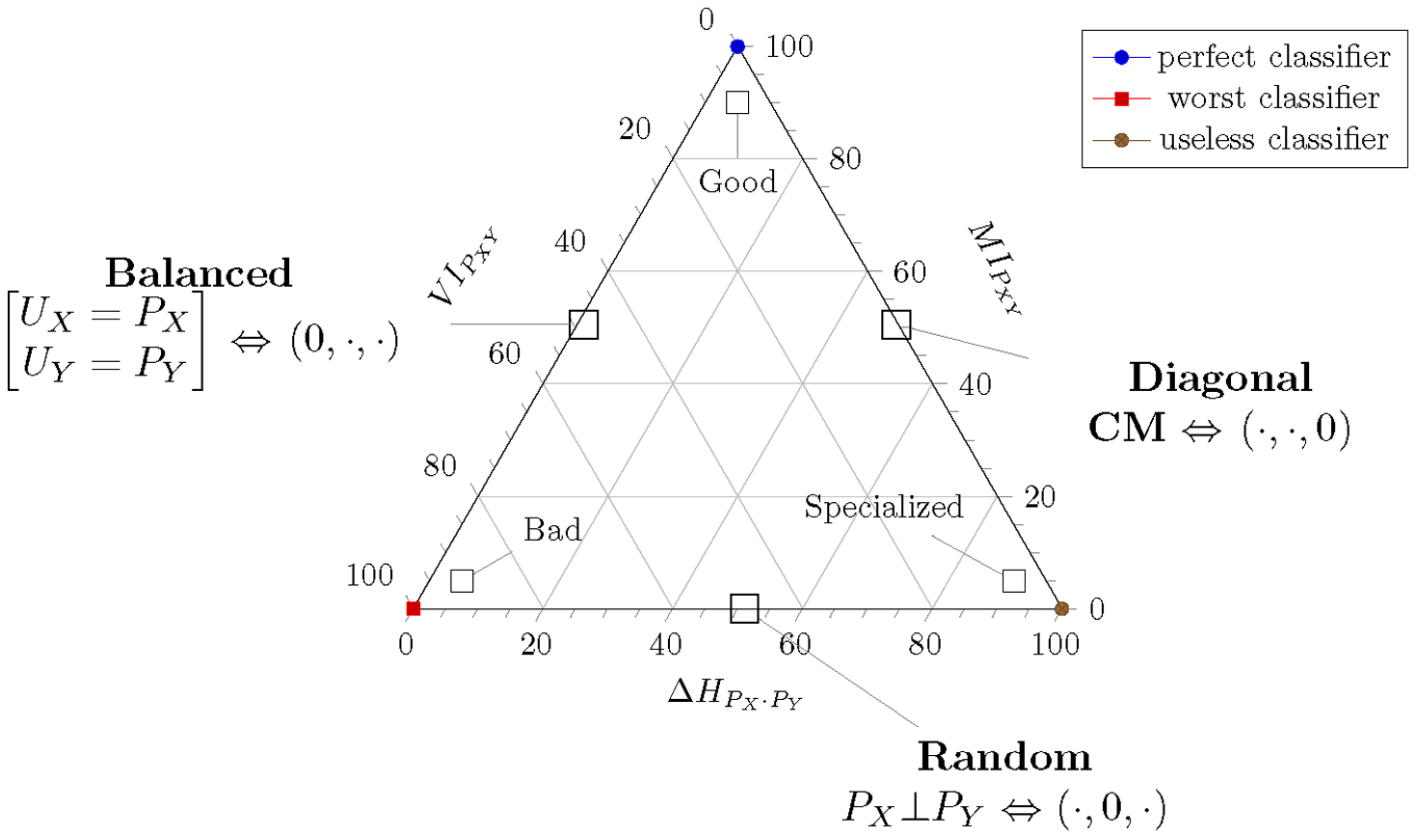
Schematic Entropy Triangle showing interpretable zones and extreme cases of classifiers. The annotations on the center of each side are meant to hold for that whole side.

The position of the coordinates of a classifier on the Entropy Triangle characterizes its performance, and we use this characterization to visually assess it indicated in Fig. 6. Classifiers at the apex or close to it obtain the highest accuracy possible on balanced datasets and transmit a lot of mutual information, hence they are the *best classifiers* possible. Those at the left vertex or close to it are dealing with balanced data but doing a bad job of utilizing it: they are the *worst classifiers*. Those at the right vertex or close to it are dealing with very easy, unbalanced data and claiming very high accuracy, yet they are not learning anything from it: they are *specialized (majority) classifier*s and our intuition is that they are the kind of classifiers that generate the accuracy paradox [16].

### The split entropy triangle

Notice that in equation (6), since both 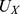 and 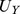 and 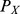 and 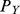 are independent as marginals of 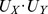 and 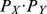, respectively, we may write:
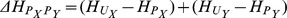


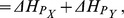
what suggests writing separate balance equations for each variable,



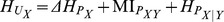


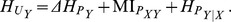



The formulae above and the occurrence of twice the expected mutual information in equation (6) suggests a different information diagram, depicted in Fig. 5(b): both variables 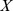 and 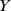 now appear somehow decoupled—in the sense that the areas representing them are disjoint—yet there is a strong coupling in that the expected mutual information appears in both 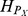 and 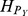. It is important to note that both decompositions can be represented in the same (split) entropy triangle as equation (6) dictates. The technique is explained in [12].

### Data

The space of 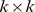 square confusion matrices, 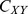 of sizes 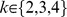 and a given number of input samples, 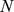, depicted in Fig. 2 was obtained by first generating every possible partition of 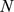 with 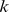 parts as input distributions 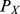, allocating 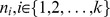 input samples in each of the input classes. In this way, the set of all possible input class distributions, from uniform 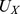 to the most skewed 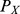, is obtained. Then, for each of the previous distributions, every possible weak composition of 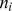 with 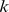 parts is produced, yielding 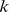 sets of all the possible distributions for each of the rows of 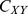. Finally, the Cartesian product of those sets produces every possible combination of rows corresponding to the selection of one element in every one of the sets. Except from row permutations —that would only amount to a reordering of the input classes— this procedure guarantees the presence of every possible 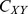.

The MEG mind reading task aims at decoding the identity of a video stimulus based on magnetoencephalography (MEG) recordings done during naturalistic stimulation [23]. In particular, subjects were exposed to video stimuli of different classes: a first category of *short* clips (6–26 s. long) with 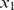 being *artificial* stimuli (screen savers showing animated shapes or text), 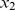 being *natural* stimuli (sceneries like mountains or oceans) and 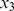 being *football* stimuli (from —European— football matches) and a second category of *long* clips (approximately 10 min. long) with 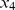 being television series (from “Mr. Bean” in particular) and 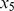 being films (from Chaplin's “Modern times”). The goal was to classify unlabeled test examples into these classes based on the MEG signal alone. The competition took place in March, 2011 and 10 participants submitted their classifiers whose confusion matrices are analyzed in this paper. The data was provided upon request from the organizers of the competition.

The MATLAB(A registered trademark of The MathWorks, Inc.) code to draw the entropy triangles in Figures 2 and 4 has been made available at: http://www.mathworks.com/matlabcentral/fileexchange/30914

## Supporting Information

Figure S1
**Heat maps of the classifiers of the MEG mind reading competition** [**23**] **.** Rows correspond to stimulus 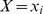 and columns to the decision 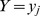 or response. Darker hues correlate with higher joint probability 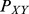. The classifier denominations obey to their position in the ranking produced by accuracy.(TIFF)

Figure S2
**Heat maps of the classifiers of the TASS competition** [**29**] **.** Rows correspond to stimulus 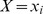 and columns to the decision 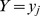 or response. Darker hues correlate with higher joint probability 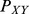. The classifier denominations obey to their position in the ranking produced by accuracy **A** Color bar represents EMA **B** Color bar represents 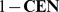 **C** Color bar represents 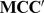.(TIFF)

Figure S3(Color online) **Entropy decomposition for the classifiers of the TASS competition (A) with the color bar representing EMA, (B)** 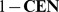 **, and (C)** 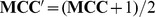.(TIFF)

Figure S4
**Heatmaps of the classifiers of the TASS competition** [**29**] **.** Rows correspond to stimulus 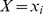 and columns to the decision 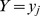 or response. Darker hues correlate with higher joint probability 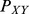. The classifier denominations obey to their position in the ranking produced by accuracy **A** Color bar represents EMA **B** Color bar represents 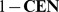] withFigures **C** Color bar represents 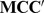.(TIFF)

Figure S5(Color online) **Entropy decomposition for MN phonetic confusion matrices (A) with the color bar representing EMA, (B)** 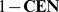 **, and (C)** 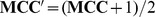 **.**
(TIFF)

File S1 **Supporting Information.** A comparison of the classical Matthew Correlation Coefficient (MCC) [28] and the Confusion Entropy (CEN) [27]—whose similarities are also explored in [10]–on three different classifications tasks: the MEG Mind Reading task already explored, the TASS sentiment analysis task [29]–both machine learning tasks—and the well-known Miller & Nicely human perceptual capability exploration task [5].
(PDF)
