# Supplementary figures and images for: 100% Classification Accuracy Considered Harmful: The Normalized Information Transfer Factor Explains the Accuracy Paradox
Source: PLoS One. 2014 Jan 10;9(1):e84217. doi: 10.1371/journal.pone.0084217 (PMC3888391; doi:10.1371/journal.pone.0084217)

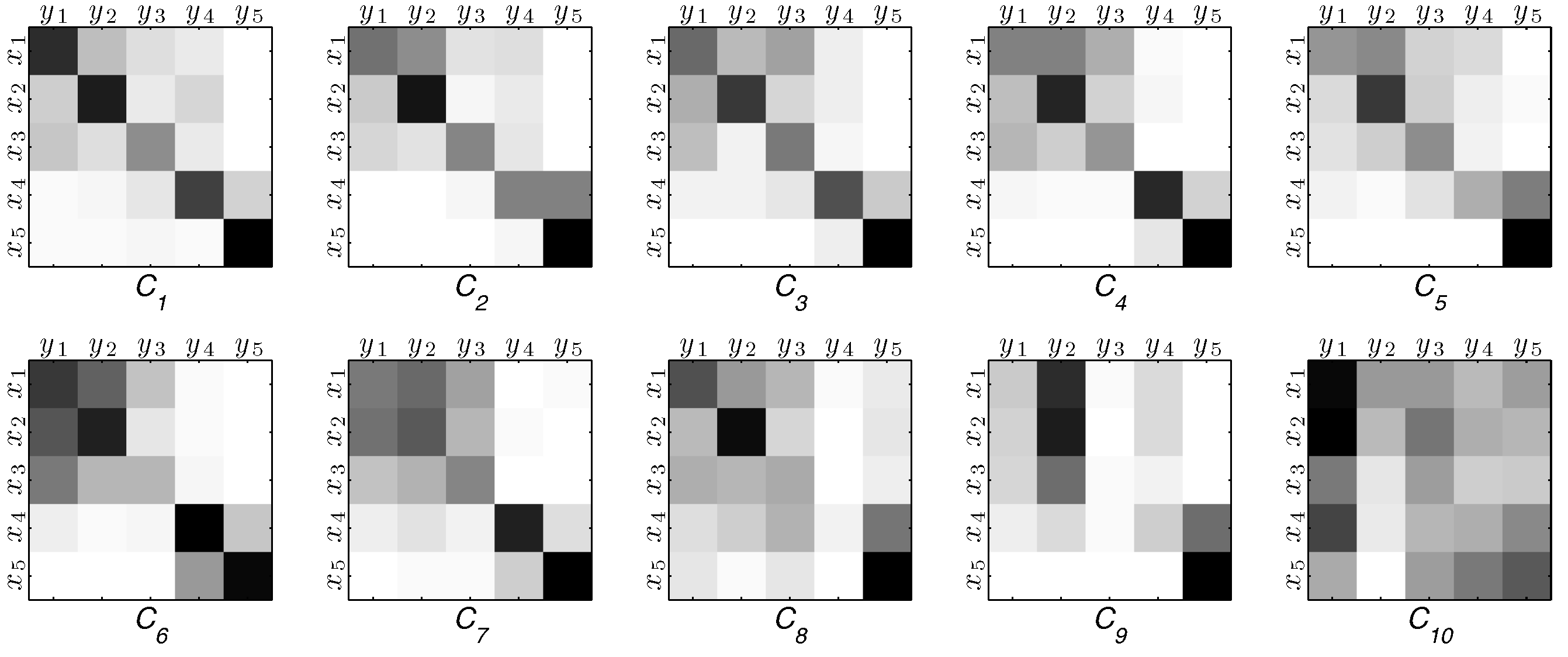

Supplement: Figure S1 — Heat maps of the classifiers of the MEG mind reading competition [23] . Rows correspond to stimulus and columns to the decision or response. Darker hues correlate with higher joint probability . The classifier denominations obey to their position in the ranking produced by accuracy. (TIFF) [file pone.0084217.s001.tiff]

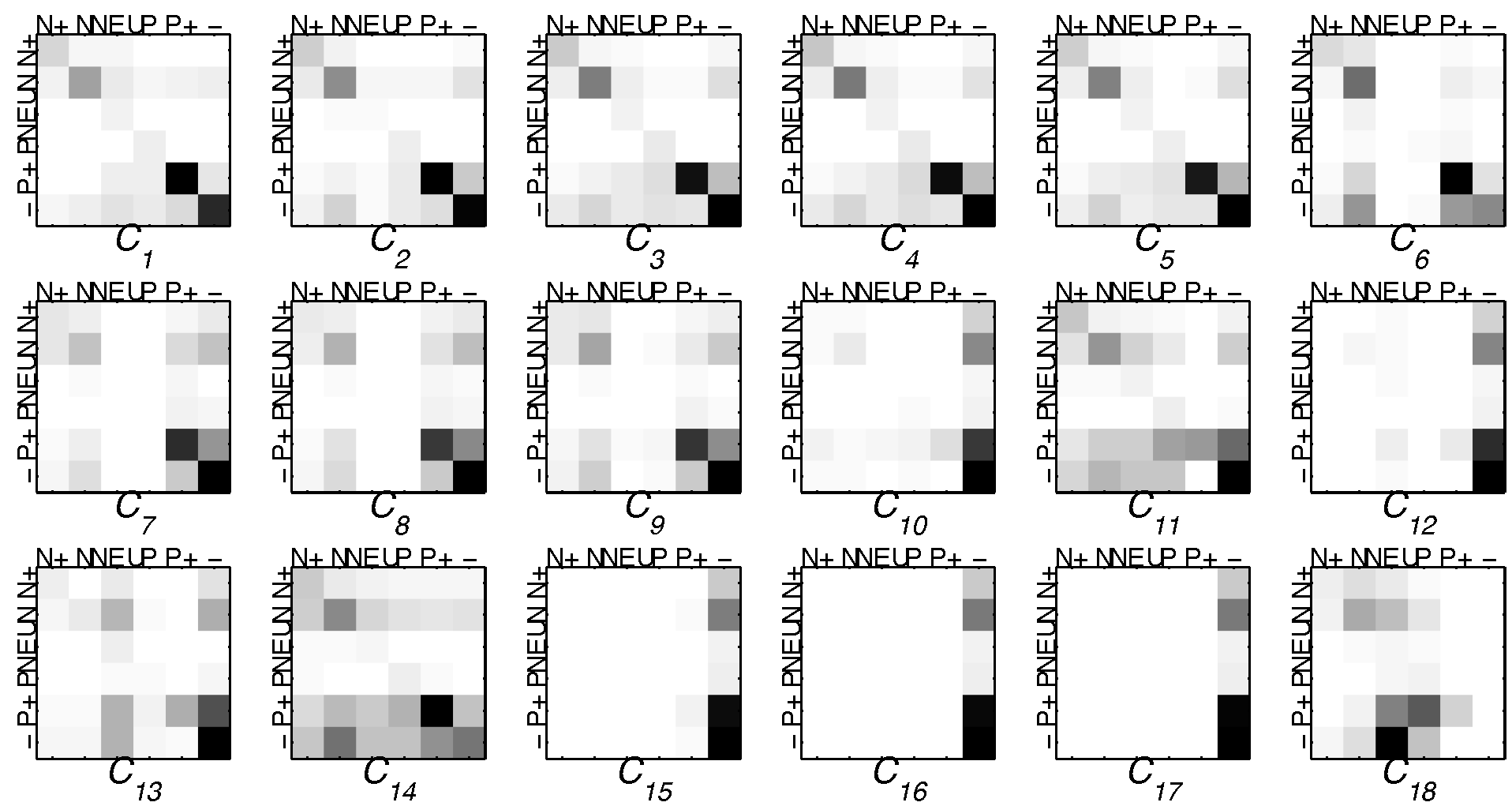

Supplement: Figure S2 — Heat maps of the classifiers of the TASS competition [29] . Rows correspond to stimulus and columns to the decision or response. Darker hues correlate with higher joint probability . The classifier denominations obey to their position in the ranking produced by accuracy A Color bar represents EMA B Color bar represents C Color bar represents . (TIFF) [file pone.0084217.s002.tiff]

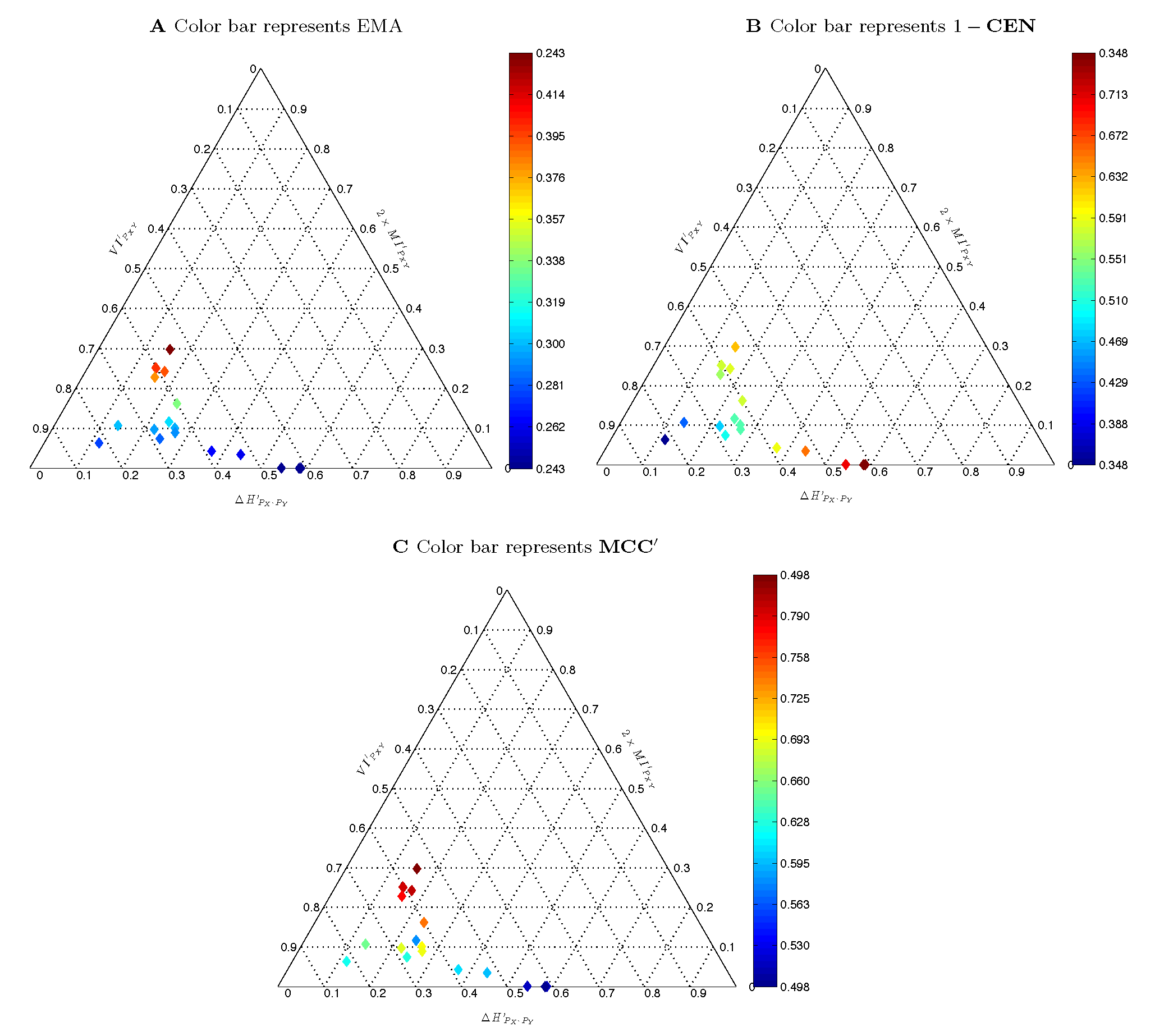

Supplement: Figure S3 — (Color online) Entropy decomposition for the classifiers of the TASS competition (A) with the color bar representing EMA, (B) , and (C) . (TIFF) [file pone.0084217.s003.tiff]

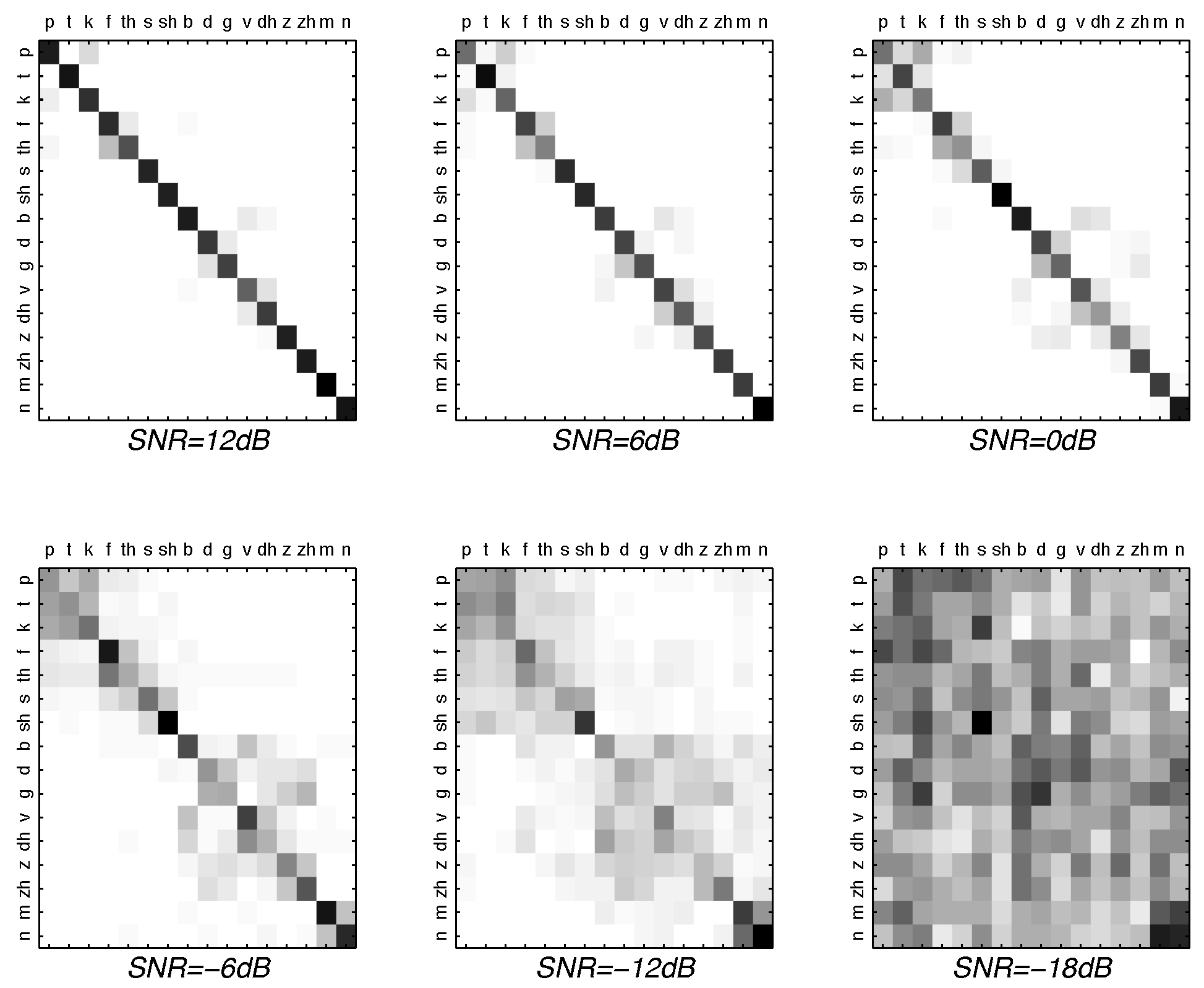

Supplement: Figure S4 — Heatmaps of the classifiers of the TASS competition [29] . Rows correspond to stimulus and columns to the decision or response. Darker hues correlate with higher joint probability . The classifier denominations obey to their position in the ranking produced by accuracy A Color bar represents EMA B Color bar represents ] withFigures C Color bar represents . (TIFF) [file pone.0084217.s004.tiff]

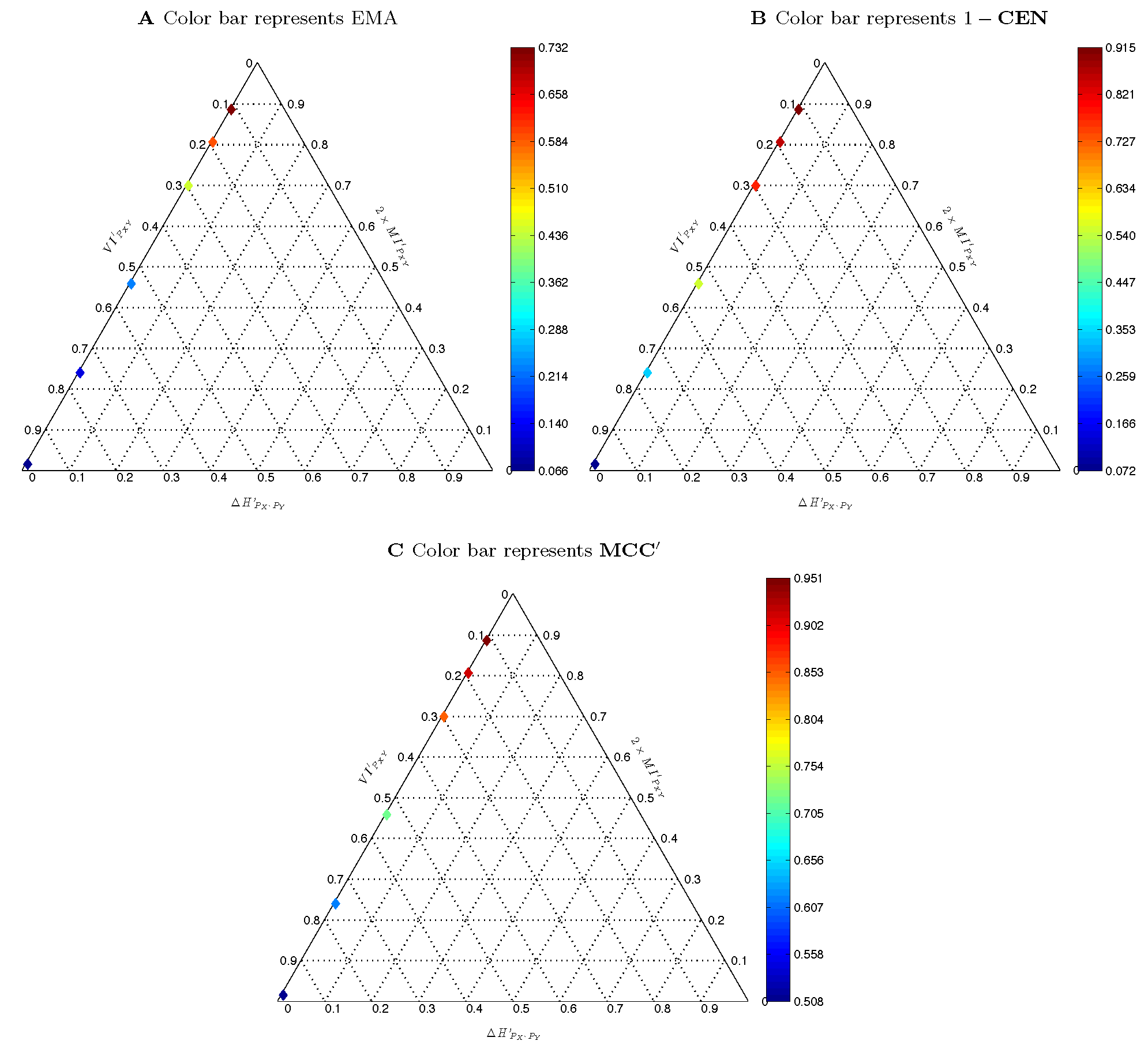

Supplement: Figure S5 — (Color online) Entropy decomposition for MN phonetic confusion matrices (A) with the color bar representing EMA, (B) , and (C) . (TIFF) [file pone.0084217.s005.tiff]
