# Supplementary material for: 100% Classification Accuracy Considered Harmful: The Normalized Information Transfer Factor Explains the Accuracy Paradox
Source: PLoS One. 2014 Jan 10;9(1):e84217. doi: 10.1371/journal.pone.0084217 (PMC3888391; doi:10.1371/journal.pone.0084217)
Supplement: File S1 — Supporting Information. A comparison of the classical Matthew Correlation Coefficient (MCC) [28] and the Confusion Entropy (CEN) [27]—whose similarities are also explored in [10]–on three different classifications tasks: the MEG Mind Reading task already explored, the TASS sentiment analysis task [29]–both machine learning tasks—and the well-known Miller & Nicely human perceptual capability exploration task [5]. (PDF) [file pone.0084217.s006.pdf]

100 % classification accuracy considered harmful: the normalized information transfer factor explains the accuracy paradox

Francisco J. Valverde-Albacete[1,*], Carmen Peláez-Moreno[2]
1 Departamento de Lenguajes y Sistemas Informáticos, Universidad Nacional de Educación a Distancia, Madrid, Spain
2 Signal Theory and Communications Department, University Carlos III Madrid, Madrid, Spain
∗ E-mail: fva@lsi.uned.es

Supporting Information

It is important to note that CEN provides a measures in the range $[0, 1]$ 1 signalling the worst and 0 the best classifiers, in the opposite order to accuracy, EMA and NIT. This is the reason why we present $(1 - \text{CEN})$ in our tables. MCC ranges in $[-1, 1]$ with -1 and 1, respectively, the worst and best possible values. Since the ET color bar can only accept positive numbers the linear transformation $\text{MCC}' = 0.5 \cdot (\text{MCC} + 1)$ was used.

Continued Analysis of the MEG Mind Reading Task

EMA and NIT factor vs. CEN & MCC. Continuing with our analysis of the MEG Mind Reading task of section *Assessing classifiers with EMA and the NIT factor*, Table S1 presents the numerical results including CEN and MCC. The heat maps of the ten classifiers of the competition can be observed in Fig. S1. Recall from our previous discussions that stimuli x_1, x_2 and x_3 belong to a particular category whilst x_4 and x_5 belong to another. The following observations are in order:

- The ranking obtained according to MCC is exactly the same as that according to accuracy, whose inability to model the phenomena under study has already been discussed in the paper.
- The ranking elicited by CEN $(C_2, C_4, C_1, C_3, C_9, C_5, C_6, C_7, C_8, C_{10})$ can be compared to that by EMA and NIT, $(C_4, C_2, C_1, C_3, C_6, C_5, C_7, C_9, C_8, C_{10})$. The first four positions are aligned with the ranking suggested by EMA and NIT with the exception of the inversion of the ordering of C_2 and C_4 which were also very close according to the latter. There is also consensus in the tail of the ranking with C_8 and C_{10} regarded as the worst classifiers.
- It is remarkable that C_9 is considered a good classifier according to CEN (the *fifth* position) while EMA and NIT relegate it to the *eighth* position and Accuracy and MCC to the *ninth* position. From Fig. S1 we can find a qualitative reason for this behavior, since C_9 inadequately privileges the y_2 and y_5 outputs over the others. This is the classical behavior of a specialized classifier, an undesirable condition that CEN fails to diagnose. An even clearer example of this behavior will be presented in *An analysis of the TASS Task*.

  This is also exemplified for CEN in [1] whose *Box 1* in *Fig. 1* shows four synthetic confusion matrices. Notice how the third one always outputs class 2 regardless of its input, evidencing the lack of any learning. Despite this, its $\text{CEN} = 0.337$ is not far from the best possible value, i.e. $\text{CEN} = 0$.

An analysis of the TASS Task

The TASS task is a Sentiment Analysis (SA) task where different human sentiment polarities and their degrees (in our case, *very positive, positive, neutral, negative, very negative, none*) need to be predicted

from Twitter messages (for more details of the experimental setup see [2, 3]). The heat maps of the eighteen classifiers in the competition can be observed in Fig. S2 and Table S2 presents the numerical results. The competition was ranked according to accuracy. ET triangles with color bars representing EMA, CEN and MCC are available in Fig. S3.

Task result evaluation. Once again we follow the procedure suggested in Section *Assessing classifiers with EMA and the NIT factor* to analyze classification performance:

1. **Use k_X to assess the effective number of classes of the data.** At $k_X = 4.114$ down from $k = 5$ for all the classifiers except one, the task is not as balanced as MEG mind reading and this may motivate some specialization-based strategies to maximize accuracy. This measure detects that participant C_{14} that did not submit answers for the full test set.

2. **Use EMA to rank classifiers.** A first group of the top five classifiers according to EMA $(C_1, C_4, C_3, C_2, C_5)$ already suggests some inversions from the ranking based on accuracy. Among the middle group of classifiers, $(C_6, C_{18}, C_{11}, C_7, C_9, C_8, C_{13}, C_{14})$, the most striking discrepancy is the seventh position that C_{18} reaches, despite being the worst classifier after accuracy. Finally, the last group $(C_{10}, C_{12}, C_{15}, C_{16}, C_{17})$ have clearly adopted a strategy based on specialization. In particular, from their confusion matrices in Fig. S2 it is evident that their output choice is most of the times *none* regardless of the input. This, for instance, makes C_{10} better ranked than others that have better learned the underlying structure of the data.

3. **Use the ET to individually assess each classifier.** As expected, EMA is perfectly aligned with increasing mutual information (right axis). The slight change in k_X of C_{14} has not caused any discrepancies between the rankings suggested by EMA and NIT. In this picture, we can distinguish the three groups of classifiers mentioned above. Notice how the five rightmost ones show the specialization trend explained above.

4. **Use the NIT factor to assess whether the population of classifiers has solved the task.** Our first observation from the ET diagram in Fig. S3A is that all the classifiers are very close to the zero Mutual Information line highlighting the fact that the task is still a big challenge. Indeed, for the top ranked classifier we have $q(C_1) = 0.284$, showing that the task has not been effectively solved by any participant.

EMA and NIT factor vs. CEN & MCC. From Fig. S3 we can observe that CEN and MCC provide very different results compared to EMA. In particular, the aforementioned specialization strategies of $(C_{10}, C_{12}, C_{15}, C_{16}, C_{17})$ make them reach the top positions according to CEN whose ranking is $(C_{16}, C_{17}, C_{15}, C_{12}, C_1, C_{10}, C_2, C_3, C_4, \ldots)$.

MCC is more robust to this situation but still provides a ranking very much like that of the accuracy, differing only from the tenth position onwards: $(C_{11}, C_{10}, C_{13}, C_{14}, C_1, C_{12}, C_{18}, C_{15}, C_{16}, C_{17})$.

The Analysis of a Human Phonetic Confusions Task

In contrast with the previous tasks solved by machine learning, we now analyze one designed to assess the human capability to discern non-contextualized consonantal sounds: the well-known experiments by Miller & Nicely [4], where aggregated human hearing confusions among sixteen consonants under different noisy conditions were examined. The heat maps of the six noisy conditions considered—as characterized by their Signal-to-Noise Ratio (SNR)—can be observed in Fig. S4, while Table S3 presents the numerical results. ET triangles with color bars representing EMA, CEN and MCC are displayed in Fig. S5.

1. **Use k_X to assess the effective number of classes of the data.** At a mean $k_X = 15.928$ down from $k = 16$ possible consonants under study, the task is almost totally balanced. Due to the settings of the experiments the human subjects will not be able to specialize. From this point of view, this is an example of a scrupulously designed experiment.

2. **Use EMA to rank classifiers.** The expected ranking, monotonically decreasing with SNR is obtained for all of the measures. From the heat maps of Fig. S4 and due to the particular ordering of the consonants presented we can observe different phonetic groups whose origins and structure have been discussed ever since. It is worth comparing these matrices, where the experimenter has highlighted the structure of the underlying perceptual phenomena, with some of the previously analyzed Sentiment Analysis and MEG Mind Reading tasks where EMA was promoting those that provide more interpretable results in spite of deviations from balanced input conditions.

3. **Use the ET to individually assess each classifier.** Our first observation from the ET diagram (Fig. S5A) is that all the classifiers are very close to the left axis implying that no specialization strategies have been used to boost accuracy since all the input classes are balanced. Also, the left axis is almost fully explored with the six noisy conditions: the bottom one corresponding to -18dB, too noisy to provide other than random choices and the top one at 12dB, close to the perfect diagonal confusion matrix that would have been represented by the apex.

4. **Use the NIT factor to assess whether the population of classifiers has solved the task.** In this case, what is actually being assessed is the human capability to discern sounds in different noise conditions. For the top ranked classifier we have $q_X(12\text{dB}) = 0.730$, showing the distance to the perfect perception of the consonants. The slight changes in k_X of the different noise conditions are not enough to produce any discrepancies between the rankings suggested by EMA and NIT.

EMA and NIT factor vs. CEN & MCC. The careful design of the Miller & Nicely experiments make all the measures analyzed valid for sorting the classifiers. However, the differences between the different evaluations can be observed in Fig. S5. Again MCC is highly correlated with accuracy, and CEN decreases more slowly as the noisy conditions worsen.

References

1. Jurman G, Riccadonna S, Furlanello C (2012) A comparison of MCC and CEN error measures in multi-class prediction. PLoS ONE 7.

2. Villena-Román J, Lana-Serrano S, Martínez-Cámara E, González-Cristobal JC (2013) TASS - Workshop on sentiment analysis at SEPLN. Procesamiento del Lenguaje Natural 50: 37–44.

3. Valverde-Albacete FJ, Carrillo-de Albornoz J, Peláez-Moreno C (2013) A proposal for new evaluation metrics and result visualization technique for sentiment analysis tasks. In: Information Access Evaluation. Multilinguality, Multimodality, and Visualization, Springer Berlin Heidelberg, volume 8138 of *Lecture Notes in Computer Science*. pp. 41-52.

4. Miller GA, Nicely PE (1955) An analysis of perceptual confusions among some English consonants. Journal of the Acoustical Society of America 27: 338–352.

5. Matthews B (1975) Comparison of the predicted and observed secondary structure of {T4} phage lysozyme. Biochimica et Biophysica Acta (BBA) - Protein Structure 405: 442 - 451.

6. Wei JM, Yuan XJ, Hu QH, Wang SQ (2010) A novel measure for evaluating classifiers. Expert Systems with Applications 37: 3799 - 3809.

7. Klami A, Ramkumar P, Virtanen S, Parkkonen L, Hari R, et al. (2011) ICANN/PASCAL2 challenge: MEG mind reading – overview and results. In: Klami A, editor, Proceedings of ICANN/PASCAL2 Challenge: MEG Mind Reading. Espoo, Aalto University Publication series SCIENCE + TECHNOLOGY 29/2011, pp. 3–19.

Supporting Figure Legends

Figure S1. Heat maps of the classifiers of the MEG mind reading competition [7]. Rows correspond to stimulus $X = x_i$ and columns to the decision $Y = y_j$ or response. Darker hues correlate with higher joint probability P_{XY} . The classifier denominations obey to their position in the ranking produced by accuracy

Figure S2. Heat maps of the classifiers of the TASS competition [3]. Rows correspond to stimulus $X = x_i$ and columns to the decision $Y = y_j$ or response. Darker hues correlate with higher joint probability P_{XY} . The classifier denominations obey to their position in the ranking produced by accuracy

A Color bar represents EMA

B Color bar represents $1 - \mathbf{CEN}$

C Color bar represents $\mathbf{MCC}'$

Figure S3. (Color online) **Entropy decomposition for the classifiers of the TASS competition (A) with the color bar representing EMA, (B)** $1 - \mathbf{CEN}$**, and (C)** $\mathbf{MCC}' = (\mathbf{MCC} + 1)/2$

Figure S4. Heatmaps of the classifiers of the TASS competition [3]. Rows correspond to stimulus $X = x_i$ and columns to the decision $Y = y_j$ or response. Darker hues correlate with higher joint probability P_{XY} . The classifier denominations obey to their position in the ranking produced by accuracy

A Color bar represents EMA

B Color bar represents $1 - \mathbf{CEN}$

C Color bar represents $\mathbf{MCC}'$

Figure S5. (Color online) **Entropy decomposition for MN phonetic confusion matrices (A) with the color bar representing EMA, (B)** $1 - \mathbf{CEN}$**, and (C)** $\mathbf{MCC}' = (\mathbf{MCC} + 1)/2$

Supporting Tables

Table S1. Accuracy $a(P_{XY})$, EMA $a'(P_{XY})$, NIT $q_X(P_{XY})$, $1-$CEN and MCC for MEG Mind Reading confusion matrices ranked by accuracy.

Classifier	$a(P_{XY})$	$a'(P_{XY})$	$q_X(P_{XY})$	$1-$**CEN**	**MCC**
C_1	0.680	0.390	0.386	0.564	0.598
C_2	0.632	0.409	0.405	0.596	0.543
C_3	0.628	0.386	0.382	0.552	0.536
C_4	0.622	0.412	0.407	0.579	0.526
C_5	0.565	0.367	0.364	0.542	0.463
C_6	0.542	0.373	0.369	0.537	0.423
C_7	0.539	0.366	0.363	0.510	0.422
C_8	0.472	0.276	0.273	0.422	0.345
C_9	0.443	0.334	0.331	0.547	0.313
C_{10}	0.242	0.208	0.206	0.172	0.052

Table S2. Perplexities, **accuracy $a(P_{XY})$, EMA $a'(P_{XY})$, NIT $q_X(P_{XY})$, $1-$CEN and MCC for TASS confusion matrices ranked by accuracy.**

Classifier	k_X	$k_{X\|Y}$	μ_{XY}	$a(P_{XY})$	$a'(P_{XY})$	$q_X(P_{XY})$	$1-$**CEN**	**MCC**
C_1	4.114	2.413	1.705	0.678	0.414	0.284	0.594	0.580
C_2	4.114	2.664	1.545	0.653	0.375	0.257	0.557	0.535
C_3	4.114	2.625	1.567	0.634	0.381	0.261	0.551	0.523
C_4	4.114	2.620	1.570	0.633	0.382	0.262	0.551	0.523
C_5	4.114	2.734	1.505	0.622	0.366	0.251	0.537	0.502
C_6	4.114	3.077	1.337	0.570	0.325	0.223	0.552	0.436
C_7	4.114	3.432	1.199	0.547	0.291	0.200	0.507	0.353
C_8	4.114	3.505	1.174	0.538	0.285	0.196	0.506	0.341
C_9	4.114	3.454	1.191	0.525	0.290	0.199	0.464	0.338
C_{10}	4.114	3.809	1.080	0.404	0.263	0.180	0.562	0.185
C_{11}	4.114	3.395	1.212	0.400	0.295	0.202	0.420	0.270
C_{12}	4.114	3.865	1.064	0.395	0.259	0.177	0.623	0.172
C_{13}	4.114	3.600	1.143	0.386	0.278	0.190	0.486	0.225
C_{14}	4.117	3.674	1.121	0.360	0.272	0.187	0.348	0.215
C_{15}	4.114	4.107	1.002	0.356	0.243	0.167	0.668	0.025
C_{16}	4.114	4.110	1.001	0.353	0.243	0.167	0.713	0.020
C_{17}	4.114	4.113	1.000	0.350	0.243	0.167	0.706	-0.004
C_{18}	4.114	3.338	1.232	0.167	0.300	0.205	0.506	0.142

Table S3. Perplexities, **accuracy $a(P_{XY})$, EMA $a'(P_{XY})$, NIT $q_X(P_{XY})$,** $1 -$ **CEN and MCC for MN phonetic confusion matrices ranked by accuracy.**

Exp.	k_X	$k_{X\|Y}$	μ_{XY}	$a(P_{XY})$	$a'(P_{XY})$	$q_X(P_{XY})$	$1-$ **CEN**	**MCC**
12dB	15.952	1.366	11.681	0.908	0.732	0.730	0.915	0.903
6dB	15.927	1.702	9.356	0.834	0.587	0.585	0.852	0.823
0dB	15.878	2.284	6.951	0.716	0.438	0.434	0.766	0.697
-6dB	15.946	4.473	3.565	0.465	0.224	0.223	0.550	0.429
-12dB	15.944	8.199	1.945	0.270	0.122	0.122	0.333	0.222
-18dB	15.919	15.260	1.043	0.078	0.066	0.065	0.072	0.017
